# Directed Assembly Network phase three launch: a round-up of success to date and strategy for the future

**DOI:** 10.1186/s13065-017-0310-4

**Published:** 2017-08-04

**Authors:** J. A. R. Rose, P. R. Raithby, C. Makatsoris

**Affiliations:** 1 School of Aerospace, Transport and Manufacturing (SATM), Cranfield University, Cranfield, MK43 0AL UK; 2 Department of Chemistry, University of Bath, Bath, BA2 7AY UK

**Keywords:** Directed assembly, Disassembly, Translation and scale-up

## Abstract

To showcase the Networks’ success during phase two (2012–2016), and to set out the strategy for phase three (2017–2019), the Directed Assembly Network held a meeting at the Royal Society in London, United Kingdom on 14 and 15 December 2016. Seventy Network members from both industry and academia attended the event. The meeting, which was used as a springboard to launch and distribute the Networks’ 2017 Roadmap to Innovation, comprised of invited talks, an advisory committee meeting, a panel Q & A session and networking.

## Introduction

The Engineering and Physical Sciences Research Council (EPSRC) Directed Assembly Grand Challenge Network began in 2010 with a 20–50 year vision: to be able to control and direct the assembly of molecules so precisely that we can develop and prepare materials with highly sophisticated and tuneable properties. Such breakthroughs in science and engineering will lead to new and advanced ways of generating clean energy, new medicines, cures and technology. The science is set out across five overlapping themes, each encompassed by directed assembly, disassembly and translation and scale-up.

The objectives of this meeting were; to showcase the network’s success to date; to outline and shape the strategy for the next 3 years; to discuss a framework for the network’s sustainability beyond that; and to launch the Network’s 2017 Roadmap to Innovation [1].

## Network successes

Paul Raithby began the meeting by describing how the Directed Assembly Network first formed and has since grown from 300 members at the start of phase 2 (2012), to over 1000 at the beginning of phase 3 (2017). This was followed by highlights and notable achievements to date.

Over £325,000 of Directed Assembly Network pump-priming, travel and seedcorn grants were awarded between 2012 and 2016 [2]. These, along with over 45 meetings during this period have led to over 80 new collaborations. Over £50M of major grants and fellowships are directly linked to and/or are supported by the network’s activities and awards [3].

The Networks’ 2017 Edition of ‘A Roadmap to Innovation’ was launched at this meeting, with printed copies distributed and made available online [1]. The roadmap describes how the network is fostering leading edge research to develop new and bespoke materials that provide the key to solving world challenges over the next 50 years. Science and engineering will help to tackle challenges such as: increasing energy demands, an ageing and growing population, antibiotic and drug resistance and a changing climate.

Roughly 25% of network members are early career researchers (ECRs), and approximately 10% are from industry. The network was praised for its continued support and strategy to support ECRs.

Presentations were given by a series ECR and mid-career researchers who had been beneficiaries of the projects that were funded as part of the network awards. The presenters demonstrated how the network has both supported their careers in general and helped them to go on to secure fellowships and/or further funding.

Several of the ECRs that have been network members from the beginning are now part of the network’s ECR advisory panel and are driving forward their own research groups.

## Future plans and strategy

Charalampos (Harris) Makatsoris kicked off day 2, outlining the plans for the network over the next 3 years and highlighted the need for the network to be innovative and how it must do more with less funding. For reference, in phase 2 the network was awarded approximately £650K spread across 4 years and in phase three £250K has been made available for a duration of 3 years.

In phase three there will be two workshops per year corresponding to the three new challenge streams: directed assembly, disassembly, translation & scale-up; resulting in two per stream within the duration of the grant. Two sandpit meetings will also take place, one for directed assembly and one for directed disassembly, both with a focus on translation and scale-up.

Two proof of concept projects will be awarded to network members to explore assembly and disassembly focusing on possible translation routes into strategic applications. These awards will be highly competitive and are expected to lead on to full Research Council UK (RCUK) grant applications.

Three ECR ‘Dreams’ meetings and three industrial consortium meetings will be held, one of each per year. It was announced that one dreams meeting is already set for mid-2017 and will be held jointly with the Dial-a-Molecule Network.

Network sustainability is a key focus point for phase 3 and as such, a strategy and framework will be developed by which to collaborate and co-fund meetings from the outset. The network will also seek to leverage funds from industry, which will enable additional pump-priming awards to be offered.

Bob Docherty (Pfizer) gave an insightful industry-perspective describing how pharmaceutical materials sciences has evolved and been shaped by academia. This was followed by Chick Wilson’s futuristic perspective on life in the year 2060; showcasing the breakthroughs and achievements gained through the network’s continuous fostering of leading edge research, echoing the 50 year goals set out in the roadmap [1].

Chick Wilson described how the networks’ vision is greater than the sum of its parts and emphasised that scientists and engineers have much to learn from biology, which has had 4 billion years to evolve and get things right, chemists on the other hand have only had 400 years so far and continue to learn!

## Q & A panel session: thoughts from attendees

The collection of talks and perspectives of directed assembly from both an academic and industry viewpoint stimulated community-led discussions and debate during the Q & A panel session, which was held before the close of the meeting on day 2.

Niek Buurma, Tim Easun, Bob Docherty, Jenny Woods and Julian Rose formed the panel for a Q & A session, which was chaired by Charalampos (Harris) Makatsoris.

The meeting participants felt that the network was very good at building bridges, including linking: industry with academia, senior researchers with ECRs and one field to another. It was also pointed out that the network tends to find things that are just ripe and succeeds in driving them forwards.

Another clear message that came across from the audience was that the breadth of the network was part of its great success, and that it is extremely important for the network to remain ‘inclusive’, open and accessible to multi-disciplinary communities.

Ideas that arose for the proposed focus of the first sandpit meeting of phase three included: disassembly, chemical stability and personalised medicine. There was also interest in holding a meeting focusing on solving ‘industry’ problems, with short, high-level talks from various industry members to inspire and motivate the multi-disciplined participants.

It was also expressed that it is very important to continue to hold meetings that combine both senior career researchers with ECRs. The emphasis of the importance of the continuation of network summer schools into phase three was another notable outcome of the discussions.

## Conclusions

The Directed Assembly Network has demonstrated both quantitative and qualitative success within the community and the UK research landscape. Through network meetings, awards and activities, tens of new collaborations have linked scientists and engineers together, across a variety of fields, who may not have otherwise have met. Overwhelmingly, the support for the network and its future is unmistakeably strong and it is clear that the continued relationship between academia and industry is highly beneficial to all parties involved. The sustainability of the network beyond phase three is crucial and strategies are either in place or are being formed to ensure its future.

## Abbreviations

EPSRC: Engineering and Physical Sciences Research Council
ECR: early career researcher
RCUK: Research Councils UK

## References

[1] Raithby PR, Makatsoris C, Woods J, Rose JAR, Price S, Wilson C, et al (2017) Directed assembly network—a roadmap to innovation. In: Raithby PR, Makatsoris C, Woods J, Rose JAR, eds. Directed assembly network 2nd edn. doi:10.6084/m9.figshare.4483502.v1

[2] Rose JAR, Makatsoris C, Raithby PR (2017) Directed assembly network awards. doi:10.6084/m9.figshare.4659151.v1. 10.1186/s13065-017-0310-4 PMC5544658 29086864

[3] Rose JAR, Makatsoris C, Raithby PR (2017) Directed assembly network—£50M grants linked. doi:10.6084/m9.figshare.4659169.v1

